# Supplementary material for: Resolving the full spectrum of human genome variation using Linked-Reads
Source: Genome Res. 2019 Apr;29(4):635–45. doi: 10.1101/gr.234443.118 (PMC6442396; doi:10.1101/gr.234443.118)
Supplement: Supplemental Material [file supp_gr.234443.118_Supplemental_longranger-2.2.zip › longranger-2.2.tar.bz2!/lr2.2-public/lib/stan/cmdstan/stan/doc/api/html/matrix__error__handling_8hpp.html]

Stan: src/stan/math/matrix\_error\_handling.hpp File Reference


|  |  |
| --- | --- |
|  | Stan  2.5.0  probability, sampling & optimization |


- Main Page
- Related Pages
- Namespaces
- Classes
- Files

- File List
- File Members

All Classes Namespaces Files Functions Variables Typedefs Enumerations Enumerator Friends Macros Pages

- src
- stan
- math

matrix\_error\_handling.hpp File Reference

`#include <stan/math/error_handling/matrix/check_cholesky_factor.hpp>`  
`#include <stan/math/error_handling/matrix/check_cholesky_factor_corr.hpp>`  
`#include <stan/math/error_handling/matrix/check_column_index.hpp>`  
`#include <stan/math/error_handling/matrix/check_corr_matrix.hpp>`  
`#include <stan/math/error_handling/matrix/check_cov_matrix.hpp>`  
`#include <stan/math/error_handling/matrix/check_ldlt_factor.hpp>`  
`#include <stan/math/error_handling/matrix/check_lower_triangular.hpp>`  
`#include <stan/math/error_handling/matrix/check_matching_dims.hpp>`  
`#include <stan/math/error_handling/matrix/check_matching_sizes.hpp>`  
`#include <stan/math/error_handling/matrix/check_multiplicable.hpp>`  
`#include <stan/math/error_handling/matrix/check_nonzero_size.hpp>`  
`#include <stan/math/error_handling/matrix/check_ordered.hpp>`  
`#include <stan/math/error_handling/matrix/check_pos_definite.hpp>`  
`#include <stan/math/error_handling/matrix/check_pos_semidefinite.hpp>`  
`#include <stan/math/error_handling/matrix/check_positive_ordered.hpp>`  
`#include <stan/math/error_handling/matrix/check_range.hpp>`  
`#include <stan/math/error_handling/matrix/check_row_index.hpp>`  
`#include <stan/math/error_handling/matrix/check_simplex.hpp>`  
`#include <stan/math/error_handling/matrix/check_size_match.hpp>`  
`#include <stan/math/error_handling/matrix/check_spsd_matrix.hpp>`  
`#include <stan/math/error_handling/matrix/check_square.hpp>`  
`#include <stan/math/error_handling/matrix/check_std_vector_index.hpp>`  
`#include <stan/math/error_handling/matrix/check_symmetric.hpp>`  
`#include <stan/math/error_handling/matrix/check_unit_vector.hpp>`  
`#include <stan/math/error_handling/matrix/check_vector.hpp>`  
`#include <stan/math/error_handling/matrix/constraint_tolerance.hpp>`  
`#include <stan/math/error_handling/matrix/validate_non_negative_index.hpp>`

Go to the source code of this file.


---

[ Stan Home Page ]

*© 2011–2014,
Stan Development Team.*
